# Waiting list eradication in serious mental illness (SMI) “secondary care” psychology: addressing an NHS blind spot

**DOI:** 10.1192/bjo.2021.809

**Published:** 2021-06-18

**Authors:** Nicola Airey, Zaffer Iqbal, Sophie Brown

**Affiliations:** 1 University of Manchester, Greater Manchester Mental Health NHS Foundation Trust; 2 Navigo Heath and Social Care CiC, University of Hull; 3 Navigo Health and Social Care CiC, University of Hull

## Abstract

**Aims:**

The poster focuses on the reduction, and eventual eradication, of waiting times within a community-based NHS psychology service in the North East of England. The poster aims to demonstrate the effectiveness of strategies implemented within a secondary care psychology service whilst examining patterns of help-seeking behaviour and treatment compliance in those waiting for therapy, and also the care needs of this cohort following a wait for services.

**Background:**

Secondary care waiting lists for psychological therapy, as highlighted by a recent British Medical Association audit, remain a so-called ‘blind-spot’ in mental health care provision and a national problem. Tackling waiting lists within this sector has been stated as a priority within the Five Year Forward View, however “core ingredients” of waiting list eradication methodologies and the components leading to such, have yet to be disseminated.

**Method:**

A historical audit and follow-up of clinical data were utilised to gather and analyse data of 208 individuals who were seen by the psychology service between October 2014 and March 2016.

**Result:**

No significant differences were found between individuals who successfully completed therapy compared to those who disengaged in regard to demographic or epidemiological variables, or mental health service input. Despite lengthy waiting times of up to 3.69 years, waiting time did not significantly impact whether someone engaged with psychological services. Any form of input from psychological services led to a significant reduction in distress, as measured by the CORE-OM. No individuals who completed therapy were re-referred for psychological input at 12-month follow-up.

**Conclusion:**

If imposed appropriately over a suitable time-frame evidence-based, effective and efficient needs-led psychological input can be provided whilst eradicating a waiting list and still remaining flexible, formulation-based and person-centred.

